# An Injectable Hybrid Gelatin Methacryloyl/Polydopamine Nanoparticle Bioink for Rapid Hemostasis Applications

**DOI:** 10.1021/acsabm.5c01762

**Published:** 2025-12-23

**Authors:** Sabrina Mai-Yi Fan, Nian-Yun Tsai, Chia-Chih Chang, Tzu-Ting Yeh, Hsin-Ling Chan, Yi-Chen Ethan Li

**Affiliations:** † Department of Chemical Engineering, 34902 Feng Chia University, Xuesi Building, No. 100, Wenhua Road, Xitun District, Taichung City 407102, Taiwan; ‡ Research Center for Cell Therapy, Department of Medical Research, 38006 National Taiwan University Hospital, No.1, Changde St., Zhongzheng Dist., Taipei City 100229, Taiwan; § Research Center for Developmental Biology and Regenerative Medicine, National Taiwan University, No. 81, Changxing Street, Da’an District, Taipei City 106038, Taiwan; ∥ Department of Applied Chemistry, 34914 National Yang Ming Chiao Tung University, Hsinchu 300093, Taiwan; ⊥ Center for Emergent Functional Matter Science, National Yang Ming Chiao Tung University, 1001, University Road, Hsinchu 30010, Taiwan

**Keywords:** gelatin methacryloyl (GelMA), polydopamine (PDA), nanoparticles, injectable ink, and hemostasis

## Abstract

Uncontrolled hemorrhage is frequently associated with high mortality. To address this challenge, engineered hydrogels have been widely investigated for hemorrhage control because of their fluid absorption and ability to promote blood coagulation. In this study, we developed injectable bioinks by combining gelatin methacryloyl (GelMA) with polydopamine (PDA) nanoparticles of various sizes. PDA nanoparticles provided abundant surface catechol groups and negative charges. We showed that GelMA hydrogels containing PDA nanoparticles smaller than 600 nm exhibited uniform porous networks. These hydrogels possessed >60% porosity and more than a 6-fold higher swelling capacity than pure GelMA hydrogels. These properties reduced blood clotting time by 2.6-fold, facilitated rapid blood absorption, and promoted fibrin network formation. In addition, the incorporation of PDA nanoparticles also increased the viscosity of the bioinks by about 3-fold when particle sizes were below 600 nm, which enhanced injectability. A dynamic *in vitro* porcine skin bleeding model showed that GelMA/PDA bioinks cross-linked with hand-held ultraviolet (UV) devices could be integrated into a portable hemostatic kit and stopped bleeding within 120 s. Furthermore, in vivo mice tail amputation and liver injury models demonstrated that hybrid GelMA/PDA hydrogels reduced clotting time by 61% and blood loss by ∼60%. These results were comparable to those of the commercial Celox hemostatic powder. In conclusion, GelMA/PDA bioinks have strong potential as injectable and cytocompatible materials for rapid hemostatic applications.

## Graphical abstract

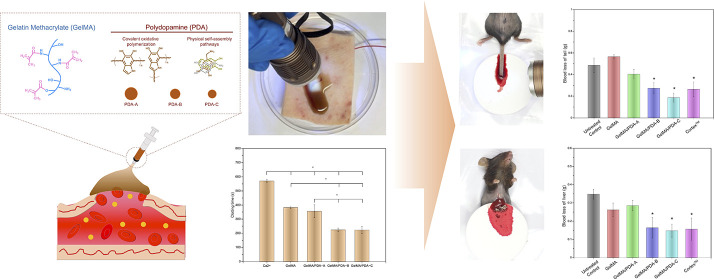

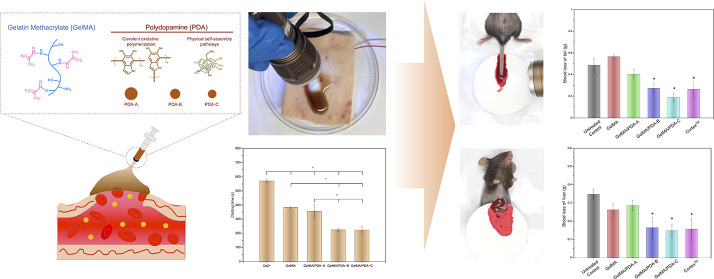

## 1 Introduction

Uncontrollable hemorrhage caused by accidents or trauma is a serious condition with high mortality rates. Conventional methods such as gauze, hemostatic powders, and pressure dressings are widely used to control bleeding, but they are sometimes not effective enough. Therefore, rapid hemostasis is essential in severe injuries and surgical procedures, which has led to the development of hemostatic biomaterials.^1^ Among hemostatic biomaterials, hydrogels have been extensively studied for hemorrhage control. For example, chitosan/gelatin hybrid gels with high fluid absorption have demonstrated good hemostatic performance in both *in vitro* and in vivo tests.^2^ Similarly, cross-linkable hydrogels undergo rapid gelation and exhibit strong adhesion, which contributes to their effectiveness in treating bleeding.^3^ In addition, chemically modified gelatin-based hydrogels with instant sol–gel transition and strong wet adhesion are easy to apply and suitable for emergency use.^4^ Compared to conventional methods, these materials offer superior fluid absorption to promote platelet aggregation and accelerate blood clotting.

Injectable biomaterials achieve rapid hemostasis by being directly applied to wounds and adapting to irregular wound shapes. These advantages offer a promising strategy for developing injectable hemostatic hydrogels. For example, injectable hydrogels as bioadhesives provide an adhesion effect to wet tissue and undergo rapid gelation, which makes them effective even under high-pressure bleeding^5,6^ In addition, these injectable hydrogels often exhibit high porosity and rapid swelling ability. These properties enable quick blood absorption and support fibrin formation and clot stabilization.^7,8^ Moreover, injectable hydrogels can be designed to incorporate bioactive components, such as calcium ions and adhesive peptides, which enhance the coagulation cascade.^8,9^ Because of their ease of use and adaptability, injectable hemostatic hydrogels are valuable in emergency care, dental procedures, and surgical applications.

Incorporating different materials endows injectable hydrogels with tunable properties, such as mechanical strength, swelling, porosity, and degradation rate.^10,11^ These characteristics play critical roles in hemostasis performance.^10^ Our previous studies have demonstrated that gelatin-based hydrogels are highly compatible with other biomaterials and can serve as functional bioinks for rapid hemostasis.^12,13^ For example, phenyl isothiocyanate-functionalized gelatin enhances supramolecular interactions of polymeric chains, which improve the injectability of photo-cross-linkable GelMA ink and increase the porosity of the GelMA hydrogel network.^12^ The increased porosity facilitates rapid blood absorption and clot formation.^12^ Additionally, we showed that combining pectin methacrylate with GelMA can form an interpenetrating polymer network and modulate the mechanical stiffness and rheological properties of injectable hydrogels. These features make the materials suitable for hemostasis applications.^13^

Recently, nanoparticles have shown great promise in hemostatic applications due to their ability to activate the coagulation cascade, enhance platelet aggregation, and promote fibrin formation.^14,15^ Their effectiveness in accelerating clotting is closely influenced by factors such as material composition, porosity, surface charge, and interactions with blood components. For instance, Marvaan and Venkatasubbu reported that calcium-modified silica nanoparticles can trigger coagulation factor activation, leading to platelet aggregation and fibrin clot development.^16^ Similarly, Abedi and co-workers demonstrated that zinc oxide-embedded chitosan sponges significantly improve porosity and surface area, thereby enhancing blood absorption, modulating thrombin activity, and promoting fibrin formation.^17^ These properties suggest that nanoparticles exhibit strong potential as additives for developing next-generation hemostatic biomaterials.

Polydopamine (PDA), inspired by mussel adhesive proteins, contains abundant functional groups such as catechols, amines, and imines.^18^ These functional groups offer numerous covalent binding sites and excellent biocompatibility for biomedical applications.^19^ Previous studies have incorporated PDA with thrombin or poly­(ethylenimine) and coated the mixtures onto membranes or fibers to improve their adhesiveness for hemostatic purposes.^2,20^ Furthermore, Ai and co-workers demonstrated that PDA nanoparticles of varying sizes provide diverse active sites, which are beneficial for catalytic and adsorption applications.^21^ Here, we engineered a PDA nanoparticle-hybrid gelatin-based injectable hydrogel for hemostatic applications. We hypothesized that combining GelMA polymers with PDA nanoparticles of varying sizes as injectable bioinks could enhance intrinsic bioactivity and internal molecular interactions, thereby accelerating blood absorption and fibrin formation for hemostasis. In this study, the chemical structure of GelMA was characterized using Fourier-transform infrared spectroscopy (FTIR) and proton nuclear magnetic resonance (1H NMR) spectroscopy. PDA nanoparticles were analyzed by FTIR, Ultraviolet–visible (UV–vis) spectrophotometry, electron microscopy, dynamic light scattering (DLS), and zeta potential analysis. Compression testing, swelling ratio, mass loss ratio, and *in vitro* burst pressure assays were used to evaluate the mechanical properties of GelMA/PDA hydrogels. Scanning electron microscopy (SEM) was used to observe the cross-linked hydrogel microstructures. For hemostatic evaluation, the rheological behavior of the hybrid bioinks was assessed using a rheometer, and cytotoxicity was tested through an *in vitro* cell viability assay. Finally, the hemostatic performance of the injectable GelMA/PDA bioinks was examined through *in vitro* clotting tests and *in vivo* mice tail amputation and liver injury models.

## 2 Materials and Methods

### 2.1 Materials

Methacrylic anhydride, gelatin from porcine skin, dopamine (DA, H8502), calcium chloride (CaCl_2_, C4901), 3-(4,5-Dimethylthiazol-2-yl)-2,5-diphenyltetrazolium bromide (MTT, M2003), trypsin (T4799), dimethyl sulfoxide (DMSO, 472301), ammonia solution (28–30%, 1.05423), and 2-hydroxy-4′-(2-hydroxyethoxy)-2-methylpropiophenone (Irgacure 2959, 410896), were purchased from Sigma-Aldrich. Dulbecco’s Modified Eagle Medium (DMEM, 10566016), and fetal bovine serum (FBS) were purchased from Thermo Fisher Scientific Inc. Mouse fibroblast cell line (L929, ATCC # CCL-1) was purchased from ATCC. Commercial porcine skin was purchased from a traditional market, commercial sheep blood was obtained from Jian Ron Ghang Co. C57BL/6 mice were purchased from the Taiwan National Laboratory Animal Center.

### 2.2 Synthesis of Polydopamine Nanoparticles and Photo-Cross-Linkable GelMA Polymers

The synthesis protocol for PDA nanoparticles was modified from a previous study.^21^ A 100 mL deionized water (DI water) and 140 mL ethanol mixture was prepared as an ethanol aqueous solution. Then, 35 mL of this solution was mixed with 0.125 g dopamine hydrochloride in a 65 mL sample vial and stirred at room temperature until fully dissolved. To synthesize PDA nanoparticles of different sizes, 0.1 mL (PDA-A), 0.25 mL (PDA-B), and 0.5 mL (PDA-C) of ammonia solution were individually added to the above mixture under mild stirring (600 rpm) at room temperature for 24 h.

The synthesis protocol for the GelMA polymer was based on our previous work.^22^ Briefly, gelatin polymer was dissolved in PBS at 50 °C to obtain a 10 w/v% gelatin solution. Then, methacrylic anhydride was gradually added to the gelatin solution drop-by-drop, and the reaction was allowed to proceed for an additional 2 h under gentle stirring. Afterward, an equal volume of PBS was mixed with the gelatin solution (*i.e.*, volume ratio = 1:1). Subsequently, the diluted gelatin solution was transferred into a dialysis membrane and dialyzed against DI water at 40 °C for 5 days. The DI water was replaced twice daily. Following dialysis, the dialyzed gelatin solution was stored at −80 °C and subsequently lyophilized for 5 days to prepare it for use.

The synthesized PDA was characterized using FTIR (PerkinElmer) and a UV–vis microplate spectrophotometer (EPOCH, BioTek). The morphology of PDA was examined using transmission electron microscopy (TEM) (JEM-1400, JEOL) and field emission scanning electron microscopy (FE-SEM) (JSM-7800F, JEOL). Additionally, the size and surface charge of PDA were determined using a particle size analyzer (PSA) (Litesizer 500, Anton Paar). The GelMA polymers and their degree of methacrylate substitution were analyzed using FTIR and ^1^H NMR (600 MHz spectrometer, Agilent Technologies).

### 2.3 Preparation of GelMA Hydrogels and GelMA/PDA Hydrogels

GelMA solutions at concentrations of 7, 10, and 15% were prepared by dissolving 70 mg, 100 mg, and 150 mg of GelMA polymers, respectively, along with either 0.05 or 0.1 g of photoinitiator, Irgacure 2959, in DPBS at 40 °C. The resulting GelMA solutions were cross-linked under UV light (800 mW/cm^2^) for 30, 60, 90, or 120 s to form hydrogels. To prepare GelMA/PDA solutions, synthesized PDA nanoparticles of different sizes were first sonicated (Q500, QSONICA, 80 W, 2 s on/1 s off), and 0.2 mL of the dispersed PDA solution was mixed with 0.65 mL GelMA solutions under stirring. The mixed GelMA/PDA solution was then photo-cross-linked under UV light (800 mW/cm^2^) for 30, 60, 90, or 120 s to form GelMA/PDA hydrogels.

### 2.4 Characterization of GelMA-Based Bioinks and Hydrogels

The rheological properties of GelMA and GelMA/PDA solutions were assessed by applying a linearly ramped shear rate from 1 to 100 s^–1^ to measure shear responses. The compressive modulus of the cross-linked GelMA/PDA hydrogels was determined using a texture analyzer (RapidTA, Horn Instruments Co., Ltd., Taiwan) at a strain rate of 20% per minute, and was calculated as the slope of the linear region corresponding to 0–5% strain. To evaluate swelling behavior, the cross-linked hydrogels were incubated in PBS, and their weight loss percentages were recorded after 7 and 14 days.

### 2.5 *In Vitro* Cytotoxicity of Hybrid Inks

L929 fibroblast cells were cultured in DMEM containing 10% fetal bovine serum (FBS), 1% penicillin, and 1% streptomycin at 37 °C in a humidified incubator with 5% CO_2_. Upon reaching approximately 80% confluency, the cells were detached using 0.5% trypsin solution, collected by centrifugation, and subcultured into fresh culture medium in a new T25 flask. To evaluate *in vitro* cytotoxicity, conditioned media obtained from GelMA and GelMA/PDA hydrogels were applied to L929 cells seeded in 24-well plates and incubated for 24 h. Subsequently, cell viability was assessed using an MTT assay. The fresh medium was replaced with conditioned medium supplemented with 10% MTT reagent. After 4 h of incubation. After 4 h of incubation, the reagent was removed, and DMSO was used to dissolve the resulting purple formazan crystals. The supernatant was then collected, and a microplate reader was used to measure the absorbance at 570 nm.

### 2.6 *In Vitro* Coagulation Time Assay

1 mL of commercial sheep blood containing 0.02 M CaCl_2_ was added to an Eppendorf tube containing GelMA or GelMA/PDA hydrogels. The mixture was gently vortexed for 10 s. The coagulation effect was evaluated by measuring the time required for visible blood aggregation.

### 2.7 *In Vitro* Burst Model

A burst pressure model was established based on previously reported protocols,^23,24^ following the ASTM F2392–04 standard method. A 4 cm × 4 cm porcine skin sheet (commercially sourced from a local food market) was presoaked in PBS prior to testing. A circular defect with a diameter of 0.8 cm was created in the center of a collagen sheet, which was then sandwiched between two 3.5 cm × 3.5 cm porcine skin sheets. The hydrogel sample was applied to seal the defect site. After gelation, the collagen sheet was removed, and the sample was mounted onto a burst pressure testing device. A syringe pump was used to generate a continuous flow of saline until rupture occurred. An absolute pressure/temperature sensor (PASCO) was employed to record the burst pressure of the GelMA and GelMA/PDA hydrogels.

### 2.8 *In Vitro* Porcine Skin Bleeding Model

The feasibility of GelMA and GelMA/PDA bioinks for blood coagulation was evaluated using an *in vitro* porcine skin bleeding model.^25^ In this model, a commercially sourced porcine skin (purchased from a local food market) was embedded with a perfusion tube delivering commercial sheep blood at a constant rate of 400 μL/min. The injectable GelMA and GelMA/PDA bioinks were then applied to the tube outlet and cross-linked *in situ* *via* UV light exposure.

### 2.9 *In Vivo* Tail Amputation and Liver Injury Models/Hemostatic Effectiveness in the Mice Model

All animal experiments were reviewed and approved by the Animal Care and Use Committee of National Taiwan University (IACUC Approval no: NTU-111-EL-00068). C57BL/6 mice were obtained from the Taiwan National Laboratory Animal Center and maintained under standard laboratory conditions (12-h light/dark cycles) with ad libitum access to food and water. The hepatic bleeding model was established according to previous studies.^26–28^ To evaluate the hemostatic efficacy of GelMA and GelMA/PDA hydrogels, male C57BL/6 mice (7–8 weeks old) were randomly assigned to experimental groups. Anesthesia was induced *via* intramuscular injection of a mixture of xylazine (Rompun, Bayer) and zolazepam (Zoletil, Virbac Laboratories) at a 1:4 ratio.

For the tail amputation model, the protocol was established based on previously reported study.^29^ Mice were first anesthetized, and a segment of the mouse tail (2–3 cm from the distal end) was amputated using a scalpel, with preweighed filter paper placed underneath to absorb blood. Immediately after amputation, the bleeding wound was treated by applying 0.5 mL of GelMA or GelMA/PDA bioinks directly to the wound. The material was then photo-cross-linked under UV light to form an *in situ* hydrogel that promoted hemostasis. Bleeding was monitored by gently lifting the tail every 30 s to check for blood flow, and the hemostatic time was recorded based on the point of complete bleeding cessation.

For the liver injury model, the protocol was established based on previously reported studies.^29–31^ Mice were anesthetized, and mice were anesthetized and disinfected prior to laparotomy to expose the liver. A 6 mm wide and 3 mm deep incision was made on the surface of the left middle lobe of the liver using a surgical knife. Immediately following injury, 0.5 mL of GelMA or GelMA/PDA bioinks were applied directly to the wound and photo-cross-linked under UV light to form a hydrogel. The bleeding status was continuously monitored and recorded every 30 s to check for blood flow until hemostasis was visually confirmed.

For comparison, a negative control group received no treatment, while a positive control group was treated with commercially available Celox hemostatic powder in both animal models. To record blood loss, preweighed filter papers were placed around the bleeding wound. Once hemostasis was visually confirmed (*i.e.*, no further bleeding or oozing), the filter papers were collected and reweighed immediately. The difference in weight was used to calculate total blood loss.

### 2.10 *In Vivo* Biocompatibility Testing

All animal experiments were reviewed and approved by the Animal Care and Use Committee of National Taiwan University (IACUC Approval no: NTU-111-EL-00068). The experimental protocol was based on previous studies.^26,28^ C57BL/6 mice were anesthetized, and the dorsal fur was shaved, and a 1 cm skin incision was made to expose the subcutaneous space. GelMA and GelMA/PDA hydrogels were implanted subcutaneously through the incision, which was closed with sutures. At 7 and 28 days postimplantation, the mice were euthanized, and the surrounding skin tissues were harvested. Hematoxylin and eosin (H&E) staining was performed on sectioned samples to evaluate potential pathological changes.

### 2.11 Statistical Analysis

Statistical analysis in this study was performed using one-way ANOVA followed by Tukey HSD test to compare data (*p < 0.05, **p < 0.01).

## 3 Results and Discussion

### 3.1 Synthesis and Characterization of GelMA Polymer and PDA Nanoparticles

Injectable hydrogels represent a promising strategy for the development of hemostatic agents due to their ability to rapidly cross-link *in situ* over irregular wound surfaces. Their hemostatic efficacy is influenced by multiple factors, including chemical characteristics (*e.g.*, surface charge, functional groups, and composite composition) and physical properties (*e.g.*, porosity and swelling behavior).^32^ In this study, we synthesized photo-cross-linkable GelMA polymers and combined them with PDA nanoparticles of three different sizes (Figure 1). The addition of PDA nanoparticles enhanced the viscosity of GelMA solutions through hydrogen bonding with the internal molecular chains.^33^ The viscous GelMA/PDA solution acts as an injectable bioink suitable for bleeding wounds. Upon UV exposure, it forms a chemically cross-linked hydrogel. Furthermore, the GelMA/PDA hydrogel features a high surface area imparted by the increased porosity from PDA nanoparticles, which in turn facilitates rapid blood absorption. In addition, bioactive sites of hydrogels provided by the functional groups on the PDA nanoparticle surface and the fibrinogen-binding motifs (*e.g.*, RGD peptides) on the gelatin polymer chains actively promote blood coagulation.^34^

**Figure 1:**
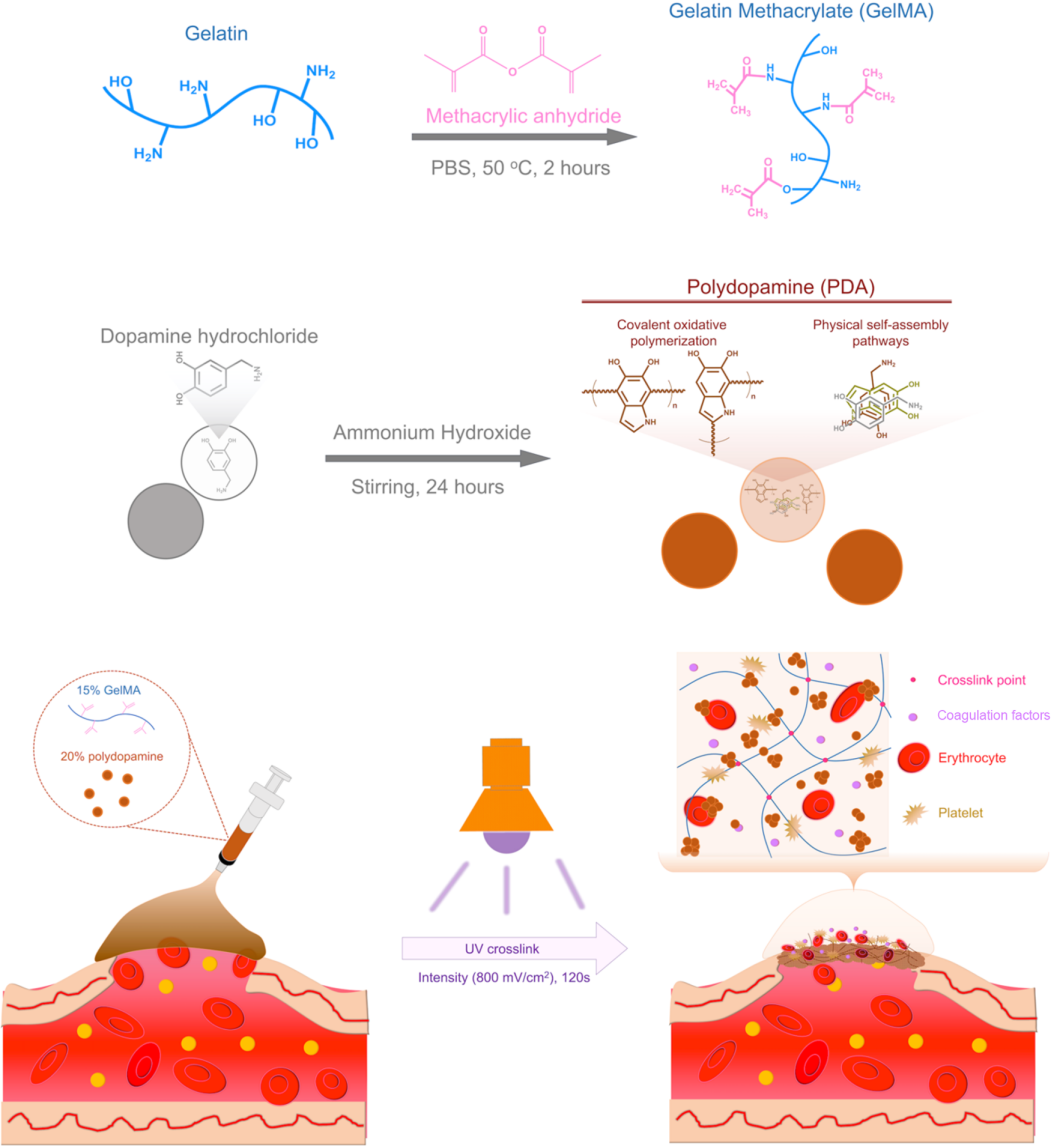
Schematic illustration of the synthesis of GelMA polymers and PDA nanoparticles and the hemostatic behavior of GelMA/PDA hydrogels cross-linked by using a UV light.

To identify the synthesized GelMA polymers and PDA nanoparticles, we first analyzed the functional groups in gelatin and GelMA using FTIR and ^1^H NMR. During GelMA synthesis, methacrylic anhydride reacts with lysine residues on the gelatin backbone. FTIR analysis showed that both gelatin and GelMA exhibited characteristic O–H, N–H, and C–H peaks at 3285, 3073, and 2935 cm^–1^ (Figure 2A). In the GelMA group, the FTIR spectra exhibited increased intensities of the C=O stretching vibration at 1630 cm^–1^, a shift in the amide II (C–N) peak to 1535 cm^–1^, and enhanced C–O–C stretching at 1082 cm^–1^. Additionally, the ^1^H NMR spectrum (Figure 2B) revealed distinct =CH_2_ doublets of methacrylate at 5.4 and 5.7 ppm. These observations confirmed successful conjugation of methacrylic anhydride onto the gelatin polymer backbone.^35^ Furthermore, we determined the degree of methacrylate substitution on GelMA polymers according to the method in our previous study.^13^ The substitution degree was approximately 47.6% substitution.

**Figure 2:**
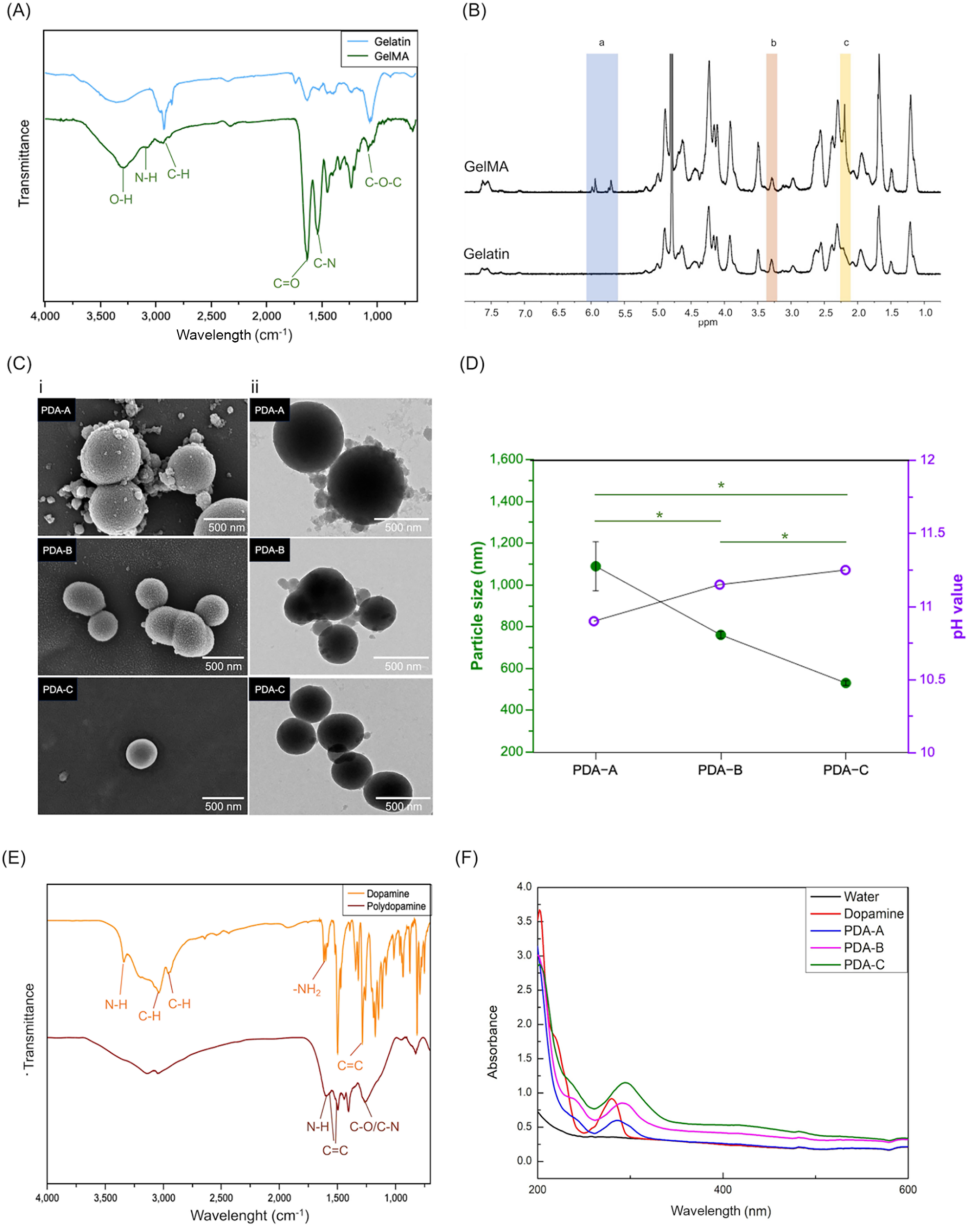
Characterization of GelMA polymers and PDA nanoparticles. (A) FTIR and (B) ^1^H NMR spectra of GelMA polymers showed characteristic relative peaks of functional groups, confirming the chemical modification of gelatin polymeric backbones with methacrylic anhydride. (C) (i) SEM and (ii) TEM images showed the morphology of PDA-A, PDA-B, and PDA-C nanoparticles. (D) DLS analysis displayed the particle sizes of PDA nanoparticles synthesized under different pH values. (*p < 0.05) (E) FTIR spectra displayed characteristic signals of dopamine and PDA functional groups. (F) UV–vis spectra showed absorption profiles of dopamine and PDA nanoparticles with different sizes.

PDA nanoparticles with three different sizes were synthesized by varying the amount of ammonia solution to modulate the pH. SEM and TEM imaging confirmed their uniform spherical morphology (Figure 2C). DLS analysis revealed average diameters of 1089  ±  118 nm for PDA-A, 761  ±  17 nm for PDA-B, and 530  ±  12 nm for PDA-C, corresponding to increasing pH levels (Figure 2D). The reduction in particle size was attributed to the elevated ammonia content, which raised alkalinity, accelerated nucleation, and limited particle growth.^36^ Figure 2E presented the FTIR spectra of dopamine and PDA nanoparticles. In the DA group, the spectra exhibited peaks corresponding to amine N–H stretching, aromatic C–H stretching, and alkyl C–H stretching at 3338, 3034, and 2931 cm^–1^, respectively. Additional peaks at 1612 and 1285 cm^–1^ were attributed to amine –NH_2_ stretching and aromatic C=C stretching vibrations.^37^ After oxidative polymerization, the PDA spectra showed broadened O–H and N–H stretching signals between 3600 and 3000 cm^–1^. A new peak at 1598 cm^–1^ was assigned to the amide N–H stretching and bending vibrations. Peaks at 1559, 1500, and 1280 cm^–1^ were associated with C=C bonds of indole functional groups and C–O or C–N stretching vibrations of the catechol moiety. These characteristic peaks related to indole and indolequinone structures confirmed the successful oxidative polymerization of PDA.^38^ Furthermore, we characterized PDA nanoparticles of the three different sizes using UV–vis spectroscopy. As shown in Figure 2F, dopamine exhibited a distinct absorption peak at 280 nm. In contrast, PDA displayed a broad absorption band from 200 to 500 nm, attributed to the formation of 5,6-dihydroxyindole units. The oxidation of dopamine quinone and subsequent polymerization of these units led to a red-shifted peak near 280 nm. Moreover, smaller PDA nanoparticles (*e.g.*, PDA-C) exhibited a larger surface area, which enhanced absorbance and red-shift behavior.^19^ These findings confirmed the successful synthesis of PDA nanoparticles.

### 3.2 Evaluation of the Mechanical Properties and Microstructure of GelMA and GelMA/PDA Hydrogels

To evaluate GelMA/PDA hydrogel formation, PDA nanoparticles of three sizes were mixed with different concentrations of GelMA. The mixtures were cross-linked into cylindrical hydrogels under UV light (800 mW/cm^2^) for 30, 60, 90, or 120 s.^22^ First, GelMA/PDA solutions with 0.5% photoinitiator were tested (Supporting Figure S1). Compared to pure GelMA, samples containing PDA showed incomplete cross-linking in the 7, 10, and 15% GelMA groups at shorter UV exposure times. This was likely due to the UV absorption and radical-scavenging properties of PDA, which reduced photoinitiator efficiency.^39,40^ To overcome this, the photoinitiator concentration was increased to 1% (Figure 3). As shown in Figure 3A, the 7% GelMA/PDA-A group showed improved cross-linking at 90 and 120 s. However, PDA-B and PDA-C still interfered with full gelation. In the 10% GelMA group, most formulations formed hydrogels, but GelMA/PDA-B and GelMA/PDA-C remained partially cross-linked at 30 s. All 15% GelMA groups formed fully cross-linked hydrogels across all exposure times. The higher GelMA concentration likely increased the number of reactive groups, resulting in enhanced cross-linking. Based on these results, 15% GelMA was selected for further experiments.

**Figure 3:**
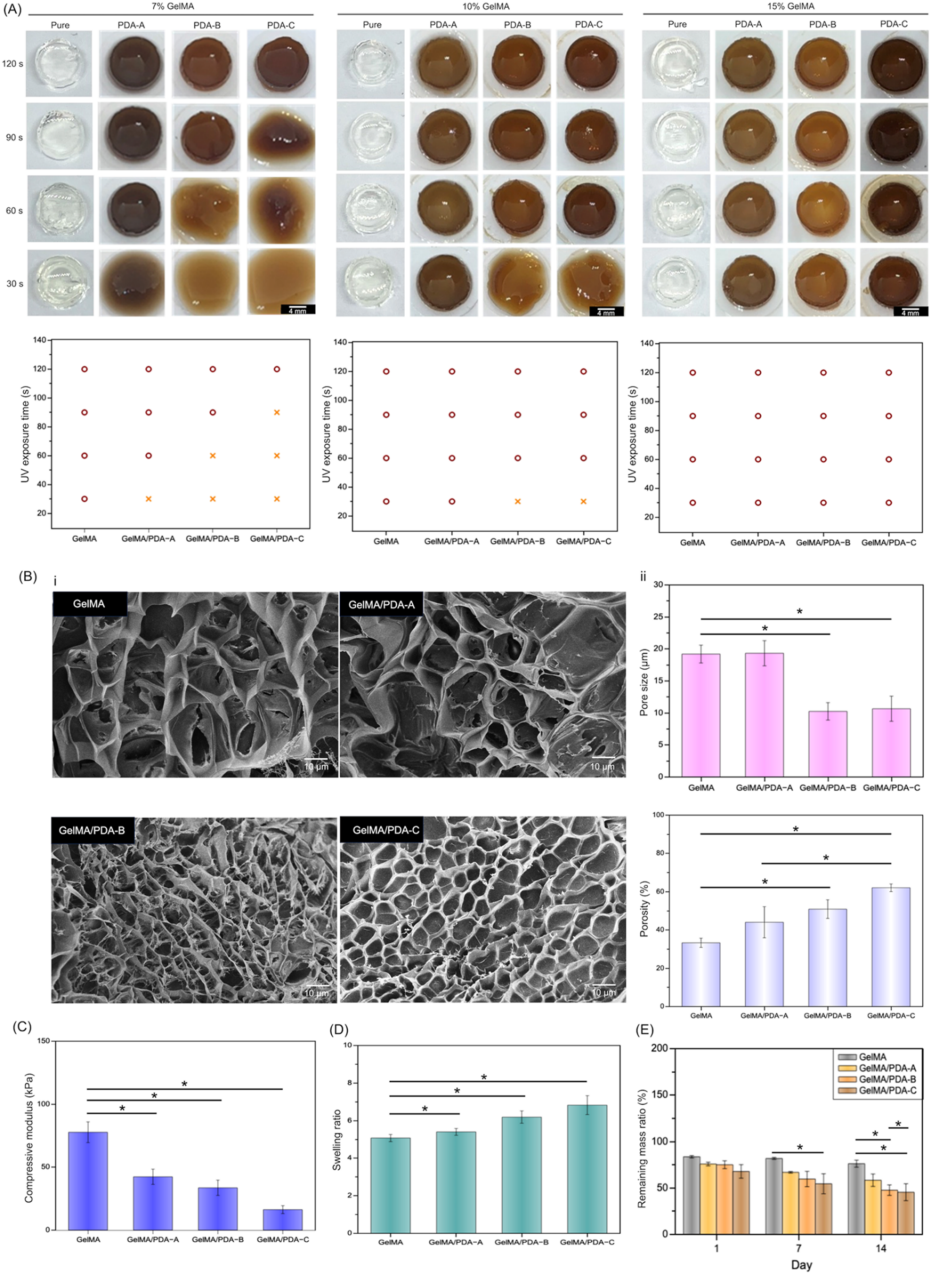
(A) Optical images show the gel formation of GelMA and GelMA/PDA solution containing 1% photoinitiator after 30s, 60s, 90s, and 120 s of UV exposure. Scale bar = 4 mm. (B-i) SEM images show the microstructure and pore size of GelMA and GelMA/PDA hydrogels. Scale bar = 10 μm. (B-ii) Pore size and Porosity of GelMA and GelMA/PDA hydrogels. (*p < 0.05) (C) Compressive modulus, (D) swelling ratio, and (E) remaining mass ratio of GelMA and GelMA/PDA hydrogels after 120 s of UV treatment. (*p < 0.05).

Figure 3B-i shows that hydrogels containing PDA-B or PDA-C nanoparticles exhibited a more homogeneous porous structure than pure GelMA and GelMA/PDA-A hydrogels. The pore sizes were 19 ± 1 μm for GelMA, 19 ± 2 μm for GelMA/PDA-A, 10 ± 1 μm for GelMA/PDA-B, and 10 ± 2 μm for GelMA/PDA-C (Figure 3B-ii). The enhanced pore uniformity in the GelMA/PDA-B and GelMA/PDA-C hydrogels was likely attributable to the smaller PDA particle size. Similarly, Figure 3B-ii showed that GelMA/PDA-B and GelMA/PDA-C hydrogels presented higher porosity (50–60%) than GelMA and GelMA/PDA-A hydrogels (30–40%).

Subsequently, the mechanical properties of the 15% GelMA/PDA hydrogels were illustrated in Figure 3C–E. As shown in Figure 3C, the compressive modulus significantly decreased with the incorporation of PDA nanoparticles. In addition, viscoelasticity was one of the key mechanical properties of the hydrogels. GelMA hydrogel was first used as an example (Supporting Figure S2­(A)) to confirm the linear elastic region. The oscillation frequency sweep test showed that both the storage modulus (*G*′) and loss modulus (*G*″) of the GelMA hydrogel remained linear, and *G*′ was higher than *G*″ when the frequency ranged from 0.1 to 100 Hz. This result indicated that the GelMA hydrogel maintained gel-like behavior within the linear viscoelastic region. Furthermore, Supporting Figure S2­(B) showed the oscillation strain sweep test conducted at a frequency of 10 Hz. When the strain reached 470%, the *G*″ exceeded the *G*′ in the GelMA hydrogel group, indicating a transition from a gel to a liquid state. The linear viscoelastic region of the GelMA hydrogel ranged from 0.01 to 470% strain. In contrast, the three GelMA/PDA hydrogels tolerated a maximum strain of only about 100%. As shown in Supporting Figure S2­(B), the GelMA/PDA-C hydrogels containing smaller PDA nanoparticles exhibited the narrowest linear viscoelastic region. Based on the results in Figure 3C, we suggested that the GelMA hydrogel exhibited higher stiffness, which resisted deformation under strain. In contrast, the incorporation of PDA nanoparticles reduced the mechanical strength of GelMA/PDA hydrogels and significantly narrowed their linear viscoelastic region. Figure 3D presented the swelling ratios of the hydrogels, which increased in the order of GelMA < GelMA/PDA-A < GelMA/PDA-B < GelMA/PDA-C. Similarly, Figure 3E shows that after immersion in the medium for 1, 7, and 14 days, the remaining mass ratios of the hydrogels decreased in the order of GelMA, GelMA/PDA-A, GelMA/PDA-B, and GelMA/PDA-C. In addition, the remaining mass ratios of all hydrogels were below 100% after 1 day. This phenomenon was likely caused by the addition of PDA nanoparticles, which interfered with the cross-linking of GelMA polymers (Figure 3A). As a result, partially un-cross-linked GelMA polymers were released from the hydrogels after 1 day of incubation. As shown in Figure 3B–E, smaller nanoparticles enhanced molecular interactions between GelMA chains and the catechol and polar functional groups of PDA, and also increased the internal porosity and uniformed the pore shape of the hydrogels. The increased porosity and pore uniformity provided more active sites, which improved water retention and promoted the formation of a more uniform cross-linked network during gelation.^32,41^ This phenomenon contributed to the observed decrease in compressive modulus and increased swelling capacity, especially in the GelMA/PDA-C group. Additionally, prolonged immersion of the hydrogels in an aqueous environment may lead to a gradual loss of molecular chain interactions within the polymer network, resulting in progressive bulk degradation and eventual mass loss.^42^

### 3.3 *In Vitro* Hemostasis and Porcine Skin Models for Examination of the GelMA and GelMA/PDA Bioinks

The hemostatic ability of GelMA/PDA hydrogels was evaluated through *in vitro* experiments (Figure 4). Following the ISO 10993–5 cytotoxicity protocol from our previous study,^25^ we tested the cytocompatibility of GelMA and GelMA/PDA hydrogels (Figure 4A). No significant differences were found between pure GelMA and GelMA/PDA, indicating that PDA nanoparticles did not induce cytotoxic effects. Because hemostatic biomaterials come into direct contact with blood, we further examined blood compatibility using a hemolysis assay. As shown in Figure 4B, the DI water group showed a red supernatant with no visible precipitate, indicating complete erythrocyte rupture. In contrast, PBS, GelMA, and GelMA/PDA groups showed clear supernatants, and all hemolysis ratios were below 5%. These results confirmed excellent blood compatibility.

**Figure 4:**
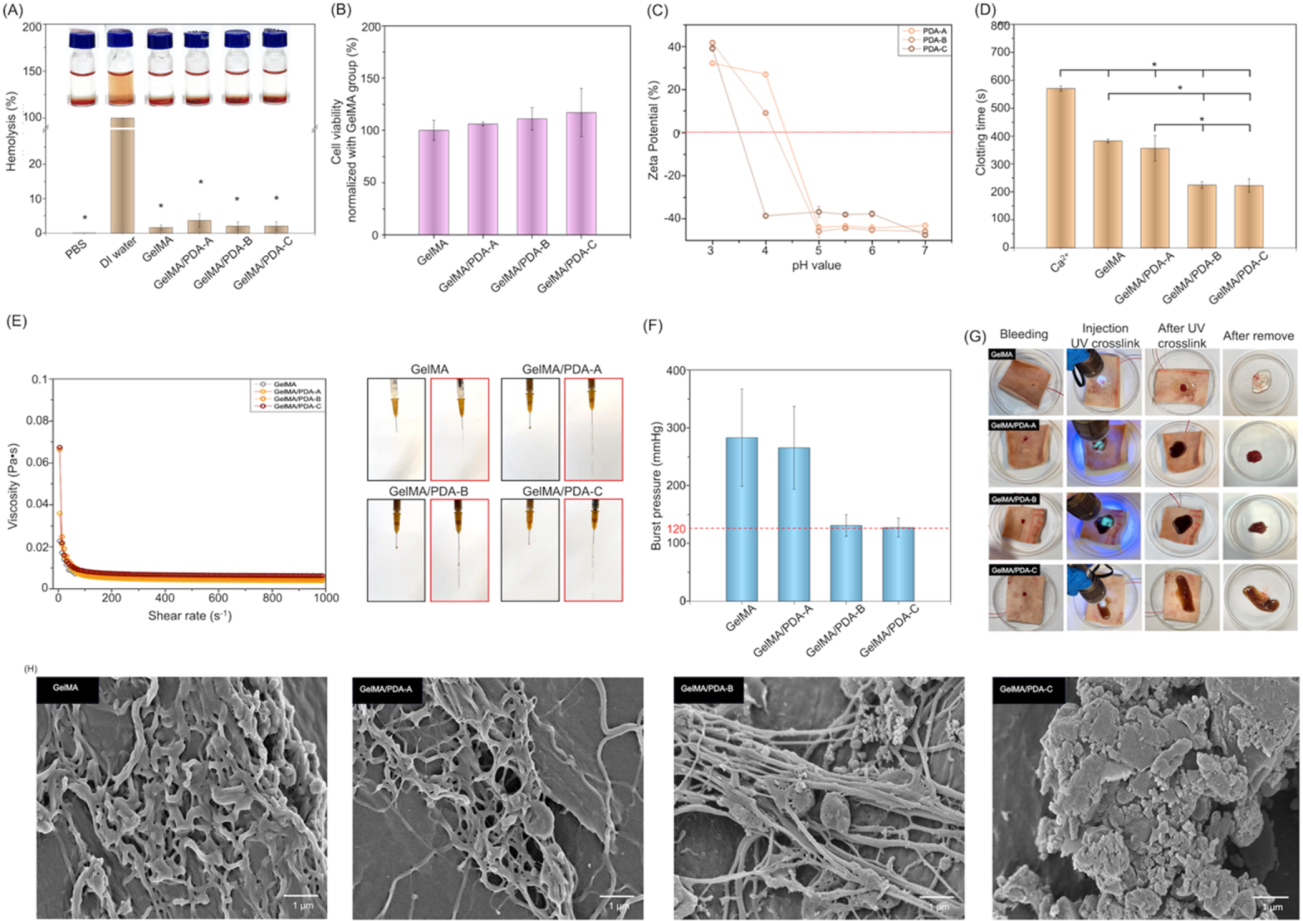
Validation of hemostatic effect of GelMA and GelMA/PDA bioinks and hydrogels. (A) Hemolysis analysis of blood incubated with GelMA and GelMA/PDA hydrogels. PBS and DI water were used as negative and positive controls, respectively. (*: Compared with DI water group, *p < 0.05) (B) Cell viability of L929 cells after 24 h of incubation with extract medium obtained from GelMA and GelMA/PDA hydrogels. (C) Zeta potential of PDA-A, PDA-B, PDA-C nanoparticles at the different pH values. (D) *In vitro* clotting time of GelMA and GelMA/PDA hydrogels upon contact with blood. (*: *p* < 0.05) (E) The size effect of PDA nanoparticles on the shear-thinning behavior of GelMA-based bioinks. (F) Burst pressure of GelMA and GelMA/PDA hydrogels after cross-linking through UV light for 120 s. (G) *In vitro* porcine skin bleeding model for mimicking the injectable GelMA and GelMA/PDA inks under blood perfusion. (H) SEM images of the fibrin formation in GelMA and GelMA/PDA hydrogels after blood clotting occurs. Scale bar = 1 μm.

Previous study has shown that negatively charged hemostatic materials are known to activate the intrinsic coagulation pathway.^43^ Therefore, we measured the zeta potential of PDA nanoparticles across different pH values. As shown in Figure 4C, PDA-A, PDA-B, and PDA-C were all negatively charged above pH 5.^44,45^ Commercial fresh sheep blood containing 0.02 M calcium ions was used as a control group (Ca^2+^ group), whose clotting time was 570 ± 10 s. Compared with the control group, pure GelMA significantly accelerated coagulation, reducing clotting time to 383 ± 7 s. This effect was attributed to the porous structure of GelMA and the bioactive segments of its polymer chains. Subsequently, incorporation of PDA nanoparticles further enhanced hemostatic performance, reducing clotting times to 356 ± 46 s for GelMA/PDA-A, 225 ± 12 s for GelMA/PDA-B, and 223 ± 24 s for GelMA/PDA-C (Figure 4D).

Next, Injectability was assessed by analyzing the rheological properties of GelMA and GelMA/PDA bioinks. As shown in Figure 4E, the apparent viscosity of all bioinks decreased with shear rate from 0 to 1000 s^–1^, confirming shear-thinning behavior. Injection tests showed that all bioinks could be extruded smoothly without clogging. Pure GelMA produced a droplet-like morphology after extrusion, whereas GelMA/PDA bioinks exhibited more gel-like behavior. This effect was attributed to PDA nanoparticles, which enhanced internal molecular interactions and increased the viscosity of GelMA/PDA bioinks. In addition, the GelMA and GelMA/PDA-C groups were used as examples to conduct an oscillation time sweep test. As shown in Supporting Figure S3, both the GelMA and GelMA/PDA-C bioinks exhibited a rapid phase transition from solid to liquid under the high strain condition (250%) and displayed mechanical recovery from the liquid to solid state during the low strain condition (0.1%). These results confirmed that GelMA/PDA bioinks could pass through the nozzle smoothly and were compatible with standard syringe systems, suggesting their potential as portable hemostatic bioinks.

Subsequently, burst pressure testing was performed to evaluate adhesive strength under blood flow (Figure 4F). Although GelMA/PDA-B and GelMA/PDA-C hydrogels showed slightly lower burst pressures, but the values in all groups exceeded normal systolic pressure (∼120 mmHg). In addition, Supporting Figure S4 showed the adhesiveness of GelMA and GelMA/PDA hydrogels. We observed that the GelMA/PDA-C group exhibited slightly higher adhesiveness than the other groups, but the difference was not statistically significant. Based on these results, the UV-cross-linked hydrogels in all groups possessed sufficient adhesive strength to remain at bleeding sites. Furthermore, we designed a preliminary *in vitro* porcine skin bleeding model to evaluate the hemostatic efficacy of GelMA and GelMA/PDA hydrogels under dynamic bleeding conditions (Figure 4G). In this model, blood was perfused at a constant rate of 400 μL/min to simulate active bleeding.^13^ GelMA and GelMA/PDA bioinks were applied to the bleeding site and cross-linked under UV light for 120 s. Both GelMA and GelMA/PDA bioinks rapidly formed cross-linked hydrogels at the bleeding site and effectively stopped blood flow. These *in vitro* findings preliminarily demonstrated the feasibility of using GelMA and GelMA/PDA bioinks as rapid hemostatic hydrogels. Notably, after the bleeding test, hydrogels in all groups were easily removed from the porcine skin without causing secondary injury.

Moreover, SEM imaging revealed fibrin network formation in GelMA and GelMA/PDA hydrogels after blood contact (Figure 4H). Compared to GelMA and GelMA/PDA-A, the GelMA/PDA-B hydrogel showed elongated fibrin fibers with entrapped erythrocytes, whereas the GelMA/PDA-C hydrogel formed a dense fibrin clot. These results suggested that PDA nanoparticles smaller than 800 nm, particularly in the GelMA/PDA-C group (530 nm, Figure 2D), provided a higher particle concentration per unit volume, leading to increased hydrogel porosity. Then, the improved porosity enhanced swelling capacity and facilitated rapid blood contact probability between blood components and surface catechol groups.^20,46^ In addition to the reasons mentioned above, a previous study reported that the complement system is a protein response network containing over 30 proteins in serum, tissue fluid, and on cell membranes.^47^ It plays key roles in inflammation, immune regulation, and coagulation. The previous study also demonstrated that PDA nanoparticles showed a concentration-dependent effect on blood coagulation.^47^ At high concentrations, their large surface area increased contact with blood components. Subsequently, the surface catechol, hydroxyl, and amino groups of PDA nanoparticles interacted with blood proteins, coagulation factors, and platelets through hydrogen bonding and electrostatic or hydrophobic forces.^48^ These interactions activated the complement system (such as C3a enzyme) and coagulation factor XII, promoting coagulation, platelet activation, and fibrin formation.^49^ Overall, these findings indicated that porous GelMA hydrogels combined with PDA-C nanoparticles exerted these synergistic effects, making them promising candidates for hemostatic applications.

### 3.4 *In Vivo* Mouse Tail Amputation and Liver Injury Models and Biocompatibility for Examination of the GelMA and GelMA/PDA Bioinks

To evaluate the practicality of GelMA/PDA bioinks, the mice tail amputation model and the liver injury model were employed as *in vivo* models.^26–28^ In the tail amputation model, an untreated group and a commercial hemostatic powder (Celox) group were used as negative and positive controls. GelMA/PDA-B, GelMA/PDA-C, and Celox showed obvious reductions in bleeding compared with the untreated, GelMA, and GelMA/PDA-A groups within 120 s (Figure 5A). Subsequently, quantitative analysis showed blood losses of 0.49 ± 0.07 g in the untreated control, 0.56 ± 0.02 g in the GelMA group, 0.40 ± 0.04 g in GelMA/PDA-A, 0.27 ± 0.05 g in GelMA/PDA-B, 0.19 ± 0.04 g in GelMA/PDA-C, and 0.26 ± 0.07 g in the Celox group (Figure 5B). These results demonstrated that GelMA/PDA-B, GelMA/PDA-C, and Celox significantly reduced blood loss by approximately 45, 61, and 47%, respectively, compared with the untreated control.

**Figure 5:**
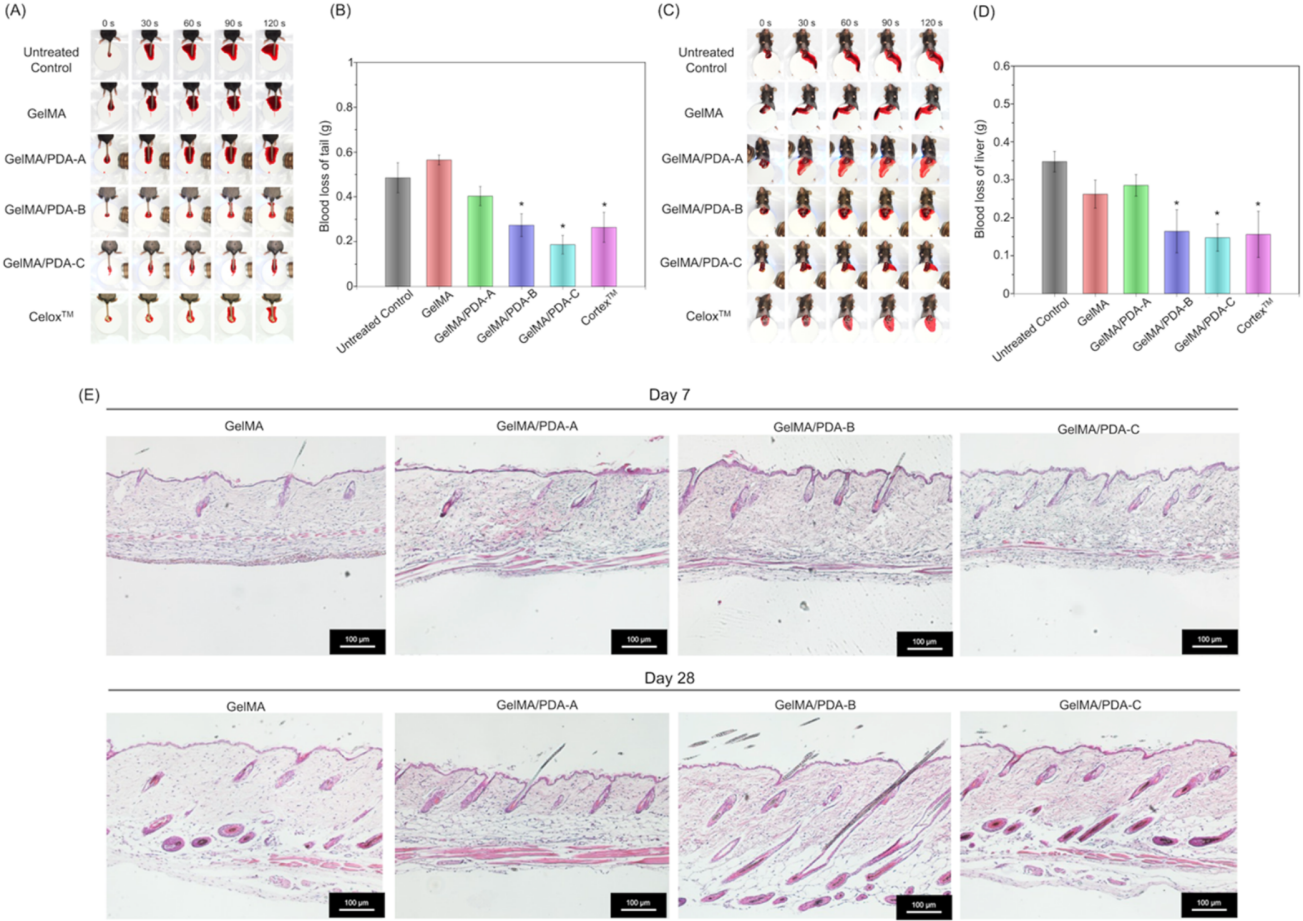
*In vivo* evaluation of the hemostatic efficacy and biocompatibility of GelMA and GelMA/PDA bioinks. Blood loss quantification in (A-B) a mouse tail amputation model and (C–D) in a mouse liver injury model. (*: Compared to the untreated control group, *:*p* < 0.05) (E) Representative H&E-stained histological images of subcutaneous tissue after 7 and 28 days postimplantation of GelMA and GelMA/PDA hydrogels. Scale bar = 100 μm.

In the liver injury model, GelMA/PDA-B, GelMA/PDA-C, and Celox also showed strong hemostatic performance, achieving bleeding control within 120 s (Figure 5C), similar to the results in the tail amputation (Figure 5B). Blood losses in GelMA/PDA-B, GelMA/PDA-C, and Celox were reduced by 53, 58, and 55%, respectively (Figure 5D). In both bleeding models, GelMA/PDA-B and GelMA/PDA-C significantly reduced blood loss compared with the untreated control. No statistical differences were found among GelMA/PDA-B, GelMA/PDA-C, and Celox, indicating that the hemostatic efficacy of GelMA/PDA-B and GelMA/PDA-C was comparable to that of the commercial powder.

Biosafety was further evaluated, as it is critical for clinical application. Although GelMA and GelMA/PDA hydrogels showed good biocompatibility in *in vitro* cytotoxicity assays (Figure 4B), their *in vivo* compatibility was tested through subcutaneous implantation in mouse dorsal tissue. As shown in Figure 5E, no significant differences were observed between GelMA and GelMA/PDA after 7 and 28 days. The hydrogels degraded gradually under physiological conditions without inducing immune responses in the surrounding tissue. Furthermore, despite potential release of PDA nanoparticles during degradation, the morphology of hair follicles remained intact, and follicular growth was unaffected at 7 days. These findings were further confirmed after 28 days. Noticeably, we did not observe any immune reaction in the *in vivo* experiment. This might have been because GelMA was derived from natural collagen and retained biocompatible motifs such as RGD sequences. These features promoted cell adhesion and enzymatic biodegradation while reducing immune recognition and chronic inflammation after implantation.^50^ In addition, PDA nanoparticles exhibited excellent biocompatibility due to their catechol and amine groups, which interacted gently with biological tissues without triggering macrophage activation.^51,52^ Moreover, PDA possessed antioxidant and radical-scavenging properties that reduced oxidative stress and suppressed inflammatory signaling.^51,52^ Collectively, the literature and our findings indicated that the GelMA/PDA hydrogels possessed favorable biosafety and biodegradability, supporting their potential as safe and effective hemostatic materials for internal organ and soft tissue bleeding.

The findings of this study demonstrated that GelMA/PDA bioinks incorporating PDA nanoparticles within a specific size range improved hemostatic performance. Among them, GelMA/PDA-C showed the greatest effect in promoting blood clotting and reducing bleeding *in vivo*. These results highlighted the critical role of nanoparticle size in modulating hemostatic behavior and suggested that sub-600 nm PDA nanoparticles are optimal for developing injectable hydrogels for rapid bleeding control. Although GelMA/PDA-C hydrogels exhibited strong performance in rapid hemostasis, they also presented limitations. The remaining mass ratio of GelMA/PDA-C hydrogels reached ∼50% after 14 days under physiological conditions. In addition, the hydrogels lacked growth factors or supplementary components to regulate cell behavior. These limitations may restrict their suitability for wound healing or tissue reconstruction after bleeding is stopped. Future studies should explore strategies to address these issues, such as chemically binding GelMA and PDA nanoparticles to slow degradation or incorporating growth factors and supplements to enhance bioactivity. In addition, PDA nanoparticles have been confirmed to exhibit many versatile functions. They possess free radical scavenging and antioxidant abilities due to their abundant phenolic functional groups.^53^ These properties make PDA nanoparticles useful additives for developing anti-inflammatory biomaterials.^51^ Moreover, the functional groups of PDA nanoparticles contain a conjugated π-electron system.^54^ This structure enables efficient photothermal conversion.^54,55^ As shown in Supporting Figure S5, the GelMA/PDA hydrogels also exhibited a photothermal conversion effect. These results suggest that GelMA/PDA hydrogels could be applied in photothermal therapy or photothermal-stimulus-based drug release systems in the future. Such approaches could expand the applications of GelMA/PDA hydrogels in wound healing and regenerative medicine.

## 4 Conclusion

In this study, we demonstrated a proof of concept for a hybrid injectable bioink composed of GelMA polymers and PDA nanoparticles for rapid hemostatic applications. Among the tested formulations, the GelMA bioink incorporating PDA nanoparticles of ∼530 nm (GelMA/PDA-C) significantly enhanced the formation of uniform porous hydrogel structures, increased swelling capacity, and improved viscosity and injectability. *In vitro* coagulation assays confirmed that this hybrid hydrogel reduced clotting time by 63%. In a dynamic porcine skin bleeding model, the bioinks exhibited excellent injectability and usability under field-mimicking conditions, supporting their potential integration into a portable hemostatic kit with hand-held UV devices. *In vivo* evaluation using mouse tail amputation and liver injury models revealed that GelMA/PDA-C hydrogels reduced blood loss by 61 and 58%, respectively, without observable immune responses. These results were comparable to those of the commercial Celox hemostatic powder. Overall, these findings highlight the GelMA/PDA hybrid bioink as a promising, cytocompatible material for field-deployable and efficient hemostasis applications.

## Supplementary Material



## Data Availability

The data supporting the findings of this study are available within the article and its Supporting Information.

## Notes

Additional experimental results including the gel formation of GelMA and GelMA/PDA solution (Figure S1); the viscoelasticity of GelMA and GelMA/PDA hydrogels (Figure S2); the oscillation time sweep results of GelMA and GelMA/PDA-C hydrogels (Figure S3); the adhessivness of GelMA and GelMA/PDA-C hydrogels (Figure S4); the photothermal effects of GelMA and GelMA/PDA hydrogels (Figure S5) (PDF)
