# Supplementary material for: An Injectable Hybrid Gelatin Methacryloyl/Polydopamine Nanoparticle Bioink for Rapid Hemostasis Applications
Source: ACS Appl Bio Mater. 2025 Dec 23;9(2):858–70. doi: 10.1021/acsabm.5c01762 (PMC12820968; doi:10.1021/acsabm.5c01762)
Supplement: Supplementary file 1 [file mt5c01762_si_001.pdf]

Supporting Information

An Injectable Hybrid Gelatin Methacryloyl/Polydopamine Nanoparticle Bioink for Rapid Hemostasis Applications

Sabrina Mai-Yi Fan[2,3 #], Nian-Yun Tsai [1, #], Chia-Chih Chang[4,5, #], Tzu-Ting Yeh[1], Hsin-Ling Chan[1], Yi-Chen Ethan Li[1, *]

[1] *Department of Chemical Engineering, Feng Chia University, Xuesi Building, No. 100, Wenhua Road, Xitun District, Taichung City 407102, Taiwan*

[2] *Research Center for Cell Therapy, Department of Medical Research, National Taiwan University Hospital, No.1, Changde St., Zhongzheng Dist., Taipei City 100229, Taiwan*

[3] *Research Center for Developmental Biology and Regenerative Medicine, National Taiwan University, No. 81, Changxing Street, Da'an District, Taipei City 106038, Taiwan*

[4]*Department of Applied Chemistry, National Yang Ming Chiao Tung University, Hsinchu, Taiwan*

[5]*Center for Emergent Functional Matter Science, National Yang Ming Chiao Tung University, 1001, University Road, Hsinchu 30010, Taiwan*

[#] *Authors are equal contribution*

[*] *Corresponding author*

Phone: +8864 24517520 ext. 3688

Email: yicli@fcu.edu.tw

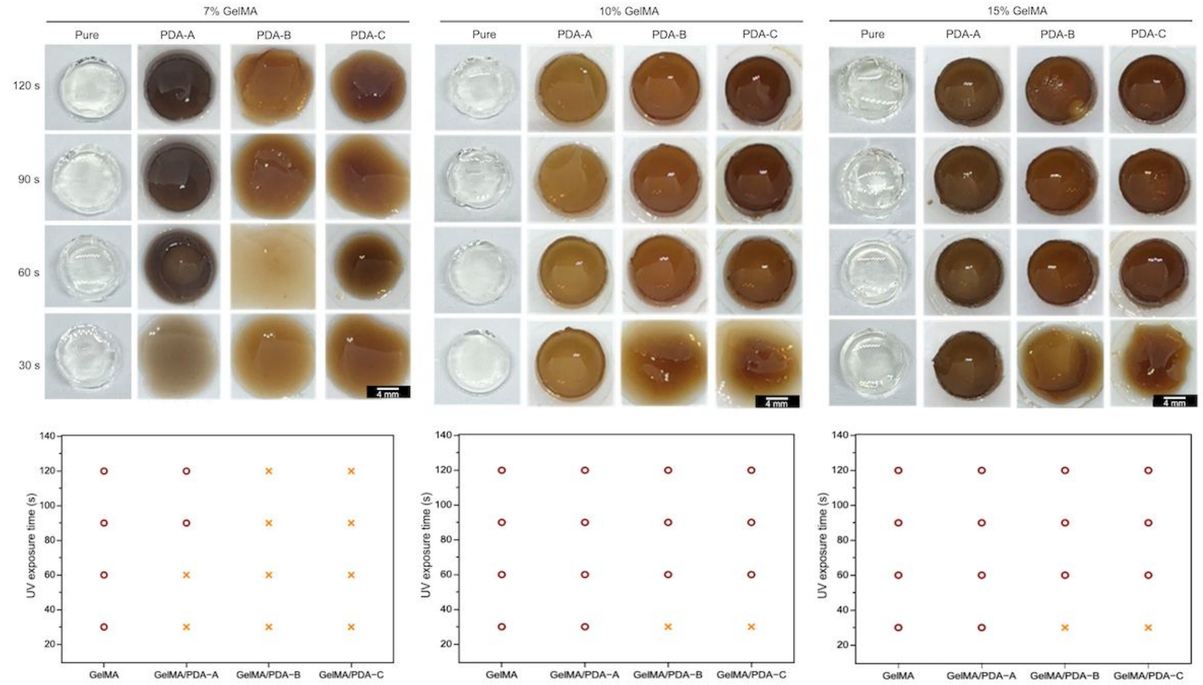

Supporting Figure S1. Optical images show the gel formation of GelMA and GelMA/PDA solution containing 0.5% photoinitiator after 30, 60, 90, and 120 seconds of UV exposure. Scale bar = 4 mm.

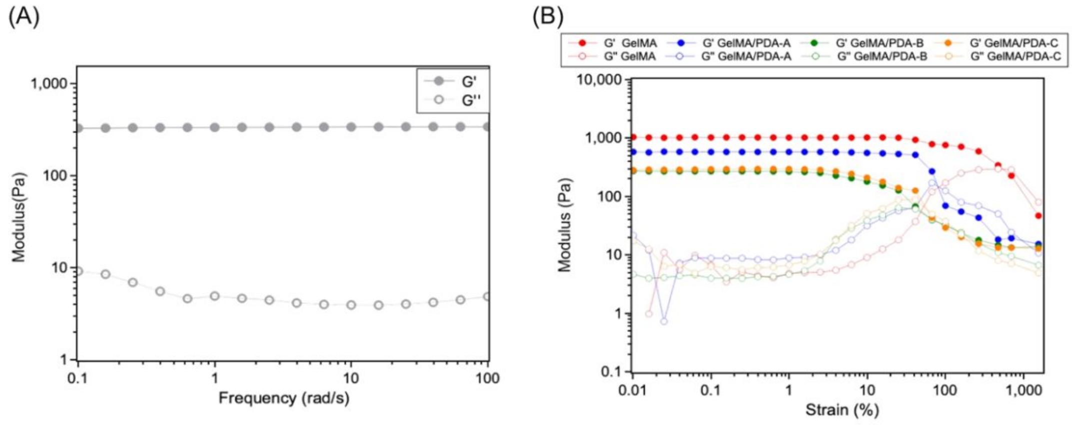

Supporting Figure S2. The viscoelasticity of GelMA and GelMA/PDA hydrogels. (a) The oscillation frequency sweep of GelMA hydrogel and (b) the oscillation strain sweep of GelMA and GelMA/PDA hydrogels.

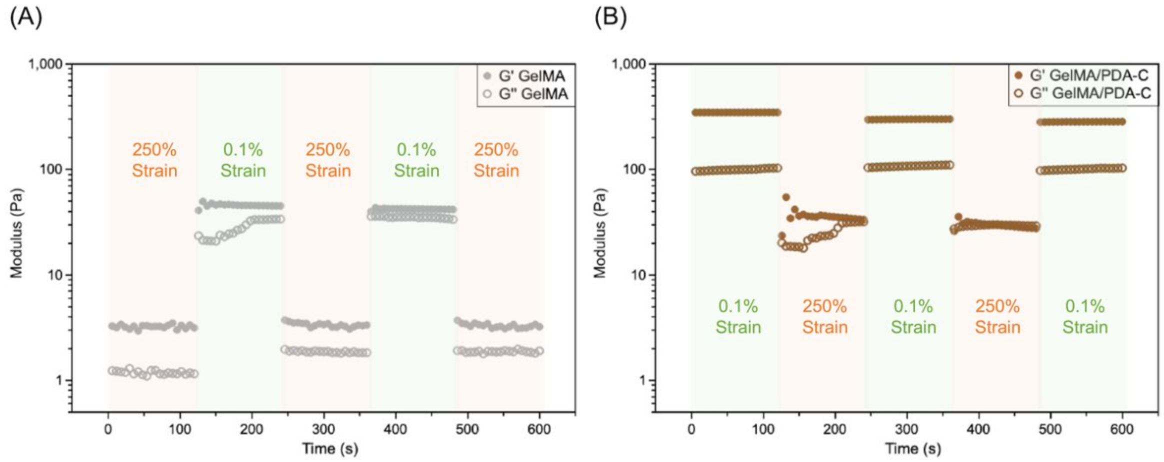

Supporting Figure S3. The oscillation time sweep results of (a) GelMA and (b) GelMA/PDA-C hydrogels under the high strain condition (250%) and the low strain condition (0.1%)

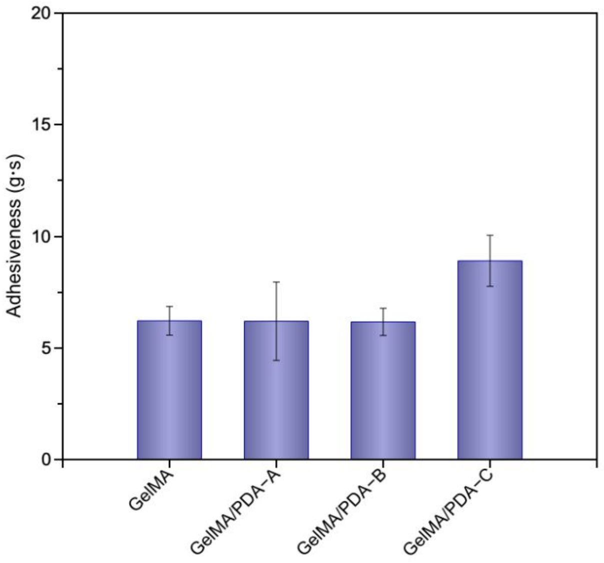

Supporting Figure S4. The adhessivness of GelMA and GelMA/PDA-C hydrogels.

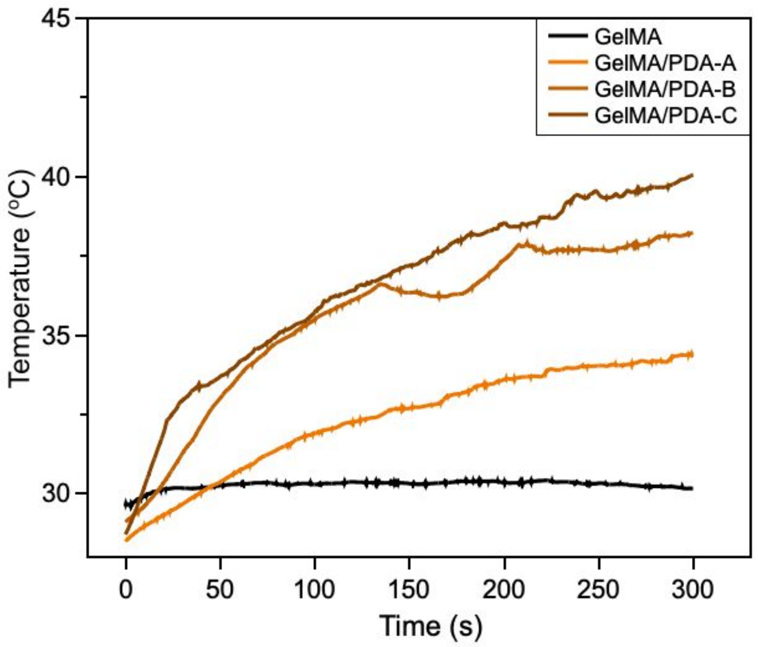

Supporting Figure S5. Photothermal effects of GelMA and GelMA/PDA hydrogels after absorbing near-infrared (NIR) light at 808 nm.
